# MEG state dynamics of sentence generation: evidence for a compensatory segmentation mechanism in healthy aging

**DOI:** 10.3389/fncom.2026.1816522

**Published:** 2026-07-01

**Authors:** Clément Guichet, Sylvain Harquel, Raouf Zouglech, Camille Lemaire, Émilie Cousin, Vincent Auboiroux, Aurélie Campagne, Monica Baciu

**Affiliations:** 1 Université Grenoble Alpes, CNRS LPNC UMR 5105, Grenoble, France; 2 Department of Neurorehabilitation, CHU Grenoble Alpes, Grenoble, France; 3 Université Grenoble Alpes, CEA LETI, Grenoble, France; 4 Department of Neurology, CHU Grenoble Alpes Université Grenoble Alpes, Grenoble, France

**Keywords:** brain state dynamics, cognition, healthy aging, hidden markov model, language, MEG, predictive processing, SENECA

## Abstract

Healthy aging is accompanied by subtle difficulties in language production. While behavioral and neuroimaging studies suggest that older adults rely on acute semantic access to maintain language abilities, the underlying neurophysiological mechanisms remain poorly understood. In particular, it is still unclear how large-scale brain dynamics reorganize to support naturalistic sentence generation with age. In this study, we investigated the spatiotemporal brain-state dynamics during covert sentence generation (GE2REC protocol) in younger and older adults using magnetoencephalography (MEG). Source-reconstructed MEG signals were analyzed using a Hidden Markov Model which identified five recurrent brain states, encompassing language-semantic, language-control, sensorimotor, and visual domains. Latent modeling was then used to relate the spectral and temporal properties of these brain states to age and language performance. Spectrally, older adults appear to redistribute oscillatory activity from sensorimotor-related states toward semantic-related states across alpha, beta, and low-gamma frequency bands. Temporally, older adults exhibit a more segmented processing sequence between semantic and sensorimotor processing which interfaces with visuo-posterior processing. These changes robustly covaried with age and better verbal fluency (semantic and lexical). Taken together, these results suggest that the older adult brain undergoes a coordinated time-frequency reorganization to support sentence production. Older individuals likely establish an embodied semantic strategy that involves a more segmented processing sequence during sentence production via visuo-posterior information processing. We speculate that this may help shape a resource-efficient, predictive route for complex cognition in older adulthood.

## 1 Introduction

The projected doubling of the world’s aging population by 2050 constitutes a major public health challenge, and consequently requires identifying the determinants of a healthy aging mind to inform preventive strategies (United Nations, 2023; WHO, 2025). Despite extensive behavioral and neuroimaging evidence, the neurophysiological mechanisms through which the aging brain dynamically supports complex cognition remain poorly understood.

Language production, as a central element of our neurocognitive system, offers a powerful framework to address this gap (Borne et al., 2024; Cui et al., 2025; Hagoort, 2023). At the behavioral level, language is increasingly used as a biomarker of the strengths and vulnerabilities in healthy and pathological aging (Benítez-Burraco and Ivanova, 2024; Mekkes et al., 2024). At the cognitive level, language involves cross-domain interactions between domain-general executive functioning, domain-specific semantic processing, and perceptuo-motor systems (Bayram et al., 2023; Bourguignon and Lo Bue, 2025; Roger et al., 2022). Therefore, language is an ideal cognitive function to investigate interacting processes, especially those drawing from domain-general and domain-specific systems in aging, such as cognitive flexibility (Endress, 2019; Guichet et al., 2026). This cross-domain architecture is further evidenced at the brain level: the classical view of a unitary “language network” is increasingly being replaced by a connectomic, whole-brain anatomo-functional description (Aliko et al., 2023; Forkel and Hagoort, 2024; Hagoort, 2019; Thiebaut de Schotten and Forkel, 2022). Accordingly, the present study focuses specifically on language production as an indicator of how aging reshapes the dynamic coordination between semantic, control, sensorimotor and perceptual systems throughout brain.

Language production is particularly vulnerable to aging (Baciu et al., 2021). Behaviorally, these difficulties manifest as increased picture-naming latencies and a higher incidence of tip-of-the-tongue states beyond midlife (Benítez-Burraco and Ivanova, 2023), especially for proper names and semantically isolated words (Bannon et al., 2024; Brown and McNeill, 1966). Sentence production is similarly affected (Peelle, 2019), especially for syntactically complex structures and tasks with high working-memory demands (Agmon et al., 2023; Kemper et al., 2004; Sung, 2015). A leading hypothesis attributes these difficulties to age-related decline in executive control (Facal et al., 2012; Rossi and Diaz, 2016), which compromise the inhibition of competing representations during lexico-semantic access (Baciu et al., 2016; Badre and Wagner, 2007; Blanco et al., 2016; Boudiaf et al., 2018; Hoffman and Morcom, 2018). For example, older adults have more difficulty resolving co-activated lexical competitors and are more susceptible to semantic interference (Federmeier et al., 2020; Hardy et al., 2022; Sung, 2015). Critically, converging evidence shows that these age-related difficulties reflect a reorganization of the underlying brain dynamics that support lexical and sentence-level production rather than a uniform loss of neural efficiency.

Notably, a large body of work suggests that the lifelong accumulation of semantic knowledge may compensate some of the language production difficulties (Boudiaf et al., 2018; Cutler et al., 2025; Gilis et al., 2025; Gollan and Goldrick, 2019; Krethlow et al., 2024). This “semantic strategy” for language production can be described at complementary levels: (i) behaviorally, Fargier and Laganaro (2023) demonstrated that older adults perform better when the word production is driven by semantic associations (i.e., inferential production) than in traditional picture naming tasks (i.e., referential production); (ii) at the brain level, the LARA-C model (Baciu et al., 2021; Baciu and Roger, 2024) highlights the recruitment of age-resilient ventral semantic pathways, supporting top-down modulation from inferior frontal to medial temporal regions to facilitate semantic memory access (Hoyau et al., 2018). Within a connectomic framework, the SENECA model offers a complementary framework that emphasizes how semantic processing also relies on embodied and perceptual systems with age (Guichet et al., 2024, 2026), echoing earlier evidence showing that sensory-perceptual enrichment can partially offset control decline in older adults (Baltes and Lindenberger, 1997; Lindenberger and Baltes, 1994; Lindenberger and Mayr, 2014). Taken together, behavioral, cognitive, and neuroimaging findings support that age-related language production reflects an adaptive reorganization rather than a uniform deterioration of our brain. Age-related decline in executive control coexists with the recruitment of semantic and sensorimotor pathways for preserving lexical access and retrieval (Guichet et al., 2026).

From a neurophysiological perspective, understanding how aging reshapes language production requires methods capable of capturing fast, transient and large-scale brain dynamics. Magnetoencephalography (MEG) is particularly suited for this purpose, as it allows the characterization of oscillatory regimes during language tasks. Relatedly, recent MEG work suggests that age-related semantic processing is grounded in predictive processing. For example, a resting-state MEG study showed that acute semantic access in older adulthood correlates with increased beta to low-gamma band dynamics (13–45 Hz) across all brain states (Guichet et al., 2026). This result is consistent with prior work linking beta-gamma synchrony to post-stimulus prediction error (Fujioka et al., 2009), and implicating gamma-band oscillations in sensory prediction errors and perceptual integration (Bastos et al., 2012; Chao et al., 2022; Jensen et al., 2014). In addition, a task-MEG study reported decreases in left temporal–parietal beta power in older adults (Zheng and Piai, 2025). These beta suppressions were interpreted as a semantic integration mechanism (Hanslmayr et al., 2009; Piai and Zheng, 2019), potentially reflecting the reactivation of content representations during syntactic and semantic context integration (Antzoulatos and Miller, 2016; Spitzer and Haegens, 2017; Zioga et al., 2023).

In sum, beta-gamma-range modulations appear as crucial substrates for establishing the hypothesized semantic strategy in older adults: beta desynchronization may facilitate the propagation of sensory prediction for semantic integration through gamma-mediated signaling (Bornkessel-Schlesewsky and Schlesewsky, 2019; Wei et al., 2025). However, the MEG evidence for this semantic strategy remains inconclusive, whether based on resting-state (Guichet et al., 2026) or picture-word interference (Zheng and Piai, 2025). This highlights the need to investigate the neural dynamics of language production in a more naturalistic fashion, embedding lexical retrieval within a continuous semantic and syntactic context.

To specify the neurophysiological mechanisms supporting the semantic strategy in aging, we adapted a naturalistic sentence generation paradigm initially performed in fMRI (GE2REC protocol; Banjac et al., 2021) to MEG. Moreover, we modeled MEG activity using a latent whole-brain dynamics approach that integrates spatio-spectral and temporal properties of the data in an unsupervised fashion. We hypothesized that sentence generation engages multiple recurrent brain states whose properties differ between younger and older adults: spectrally, we expected age-related modulations in the beta and low-gamma oscillatory regimes, correlating with a semantic strategy for sentence generation. Temporally, we expected a distinct cyclical architecture between brain states with age, reflecting how the processing stages of sentences production are re-organized to establish the semantic strategy.

## 2 Materials and methods

### 2.1 Participants

MEG recordings were acquired from 22 healthy participants divided into a younger adult group (*N* = 12, 6 females, 6 males, mean age = 28.3 yo, *SD* = 4.2) and an older group (*N* = 10, 4 females, 6 males, mean age = 65.2 yo, *SD* = 6). All participants were native French speakers, had normal or corrected-to-normal vision, and were right-handed (laterality was assessed using Edinburgh Handedness Inventory; Oldfield, 1971). All participants provided written informed consent prior to participation. The study was approved by the local ethics committee of CHU Grenoble Alpes (CPP MEG-AGING n° *38RC18.294*, CHUGA).

Participants had no history of neurological disease, no major psychiatric disorders, and met a cognitive inclusion criterion defined by a Mini-Mental State Examination (MMSE) score greater than 25/30 (Kalafat et al., 2003). Anxiety and depressive symptoms were assessed using the HAD scale (Snaith and Zigmond, 1986) and added as covariates in statistical models. Cognitive abilities were evaluated using standardized measures for language, including vocabulary with the Mill-Hill test (Deltour, 1993), lexical and semantic fluency (Cardebat et al., 1990), and executive functions with the BREF (Dartinet and Martinaud, 2005) and the TMT (Tombaugh, 2004) (see Table 1).

**Table 1:** Demographic and neuropsychological characteristics of the participants.

ID	Age and gender			TIV	MMSE	BREF	HAD-A	HAD-D	TMT-A	TMT-B	VF semantic	VF lexical	Mill-Hill (A + B)
*S1*	*Y*	*28*	*F*	1,530,42	30	19	6	0	18	28	37	17	80
*S2*	*Y*	*26*	*F*	1,417,24	30	18	5	1	16	39	38	36	72
*S3*	*O*	*70*	*M*	1,440,27	29	18	9	2	30	60	41	19	86
*S4*	*O*	*59*	*F*	1,470,88	30	18	5	0	27	41	31	27	78
*S5*	*O*	*75*	*F*	1,353,45	29	18	5	3	35	73	27	19	74
*S6*	*Y*	*26*	*M*	1,480,66	29	18	6	4	16	34	41	33	78
*S7*	*Y*	*39*	*F*	1,409,18	30	18	11	2	12	28	46	25	80
*S8*	*O*	*58*	*M*	1,400,15	28	18	1	2	18	41	26	28	74
*S9*	*Y*	*25*	*F*	1,361,40	30	18	6	5	14	23	36	31	73
*S10*	*O*	*61*	*M*	1,482,63	29	18	6	0	24	36	41	24	82
*S11*	*Y*	*25*	*M*	1702,60	29	18	7	7	37	34	35	30	64
*S12*	*O*	*66*	*M*	1,633,97	29	19	5	0	20	44	30	24	61
*S13*	*O*	*63*	*F*	1,337,13	30	20	7	0	21	58	37	27	79
*S14*	*O*	*60*	*F*	1,413,97	28	19	4	0	35	112	54	33	83
*S15*	*Y*	*25*	*M*	1,582,01	30	18	2	3	19	30	51	41	80
*S16*	*O*	*67*	*M*	1,538,63	30	19	7	6	25	43	32	25	78
*S17*	*Y*	*28*	*M*	1,589,39	30	19	5	1	19	32	33	22	79
*S18*	*Y*	*33*	*M*	1,399,34	29	18	6	4	15	54	19	31	80
*S19*	*O*	*73*	*M*	1,491,61	29	18	1	1	36	62	33	26	75
*S20*	*Y*	*28*	*F*	1,582,49	30	19	14	4	21	37	33	25	73
*S21*	*Y*	*26*	*F*	1,302,83	29	18	3	2	17	37	29	26	77
*S22*	*Y*	*31*	*M*	1,427,94	29	18	7	0	29	86	37	26	74

Barred subjects (S4, S5, S14) were excluded for statistical analysis due to strong aperiodic activity compared to the other subjects (see section 2.5). Y, Younger; O, Older; F, Female; M, Male; TIV, Total Intracranial Volume; VF, Verbal Fluency.

### 2.2 Experimental protocol

The GE2REC (Generation, Recognition, Recall) protocol, initially developed for fMRI (see Banjac et al., 2021 for details) was adapted to MEG to investigate the neural dynamics of the language-memory interaction. We focused our study on analyzing the process of “Sentence Generation” because it simultaneously involves lexical retrieval, semantic integration, and syntactic planning, making it particularly well suited for investigating the neural dynamics of language in a naturalistic fashion.

**Figure 1:**
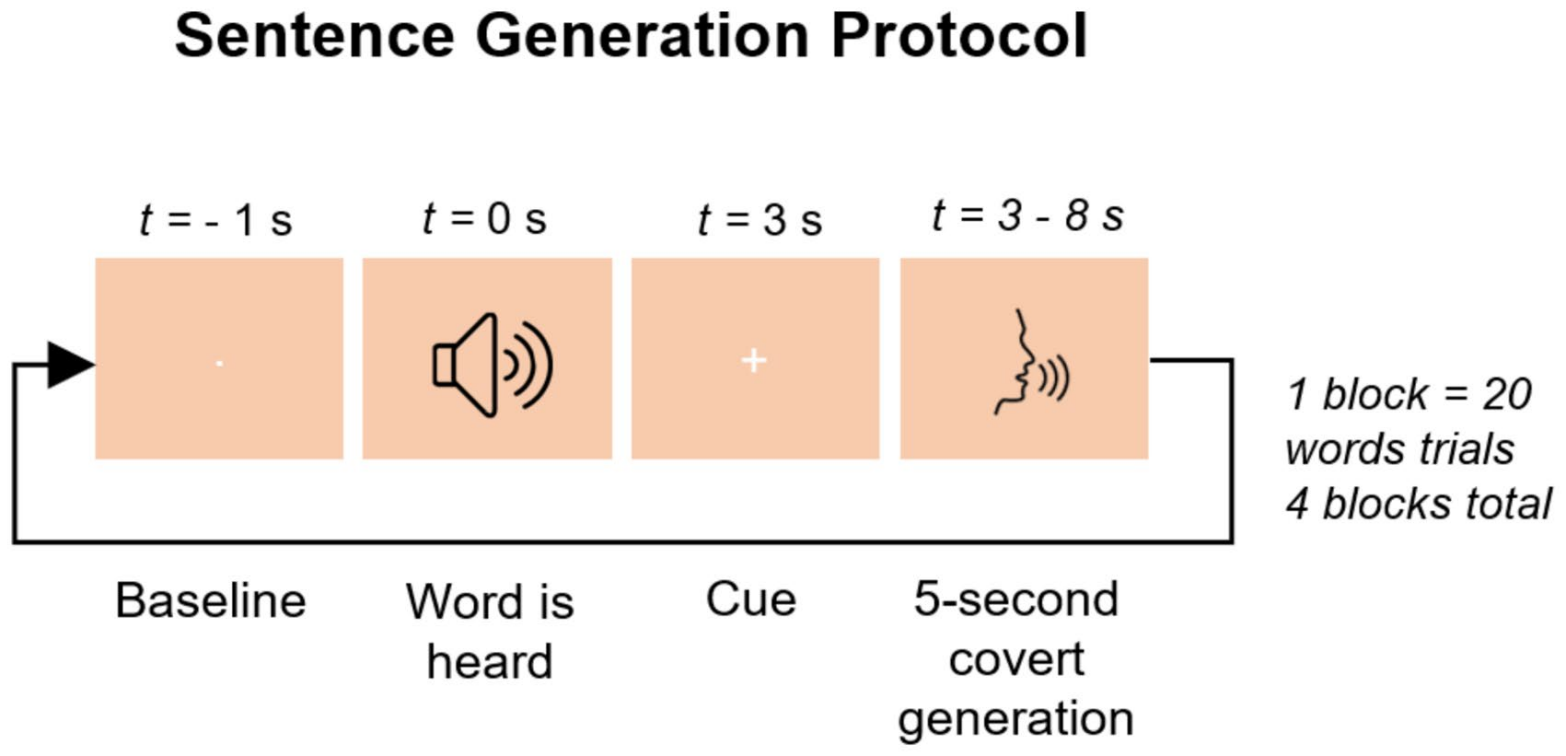
Schematic of the experimental protocol. Stimuli (cue words) were presented in the auditory modality, then sentences using the cue word were covertly generated for 5 s. We focused on the covert sentence generation period in this study.

Figure 1 illustrates the block design, which consisted of 40 randomized trials of 9 s for a total of 6 min per block. A block alternated between the task (20 trials with words) and control (20 trials with pseudowords) conditions. The entire protocol had 4 blocks with a resting period in between during which participants fixated a cross on a black screen. During the task condition, participants covertly generated short sentences for 5 s (from *t* = 3 s to *t* = 8 s) after hearing a word at *t* = 0 s. Covert sentence generation allowed us to efficiently probe sensorimotor coordination, minimizing the articulatory-motor artifacts and external feedback while still preserving a temporal structure equivalent to the one observed in overt speech (Mantegna et al., 2026). Words were taken from the French standardized naming test DO80 (Deloche and Hannequin, 1997). During the control condition, participants listened to and repeated a pseudoword (/*mistoudin*/) covertly instead of generating sentences.

### 2.3 MEG data acquisition and processing

#### 2.3.1 Acquisition

MEG data were collected in magnetically shielded room in a 306-channel Vectorview MEG system (Elekta Neuromag), 102 magnetometers, and 204 orthogonal planar gradiometers (Clinatec Neuroimaging Facility). Data were sampled at 1 kHz.

**Figure 2:**
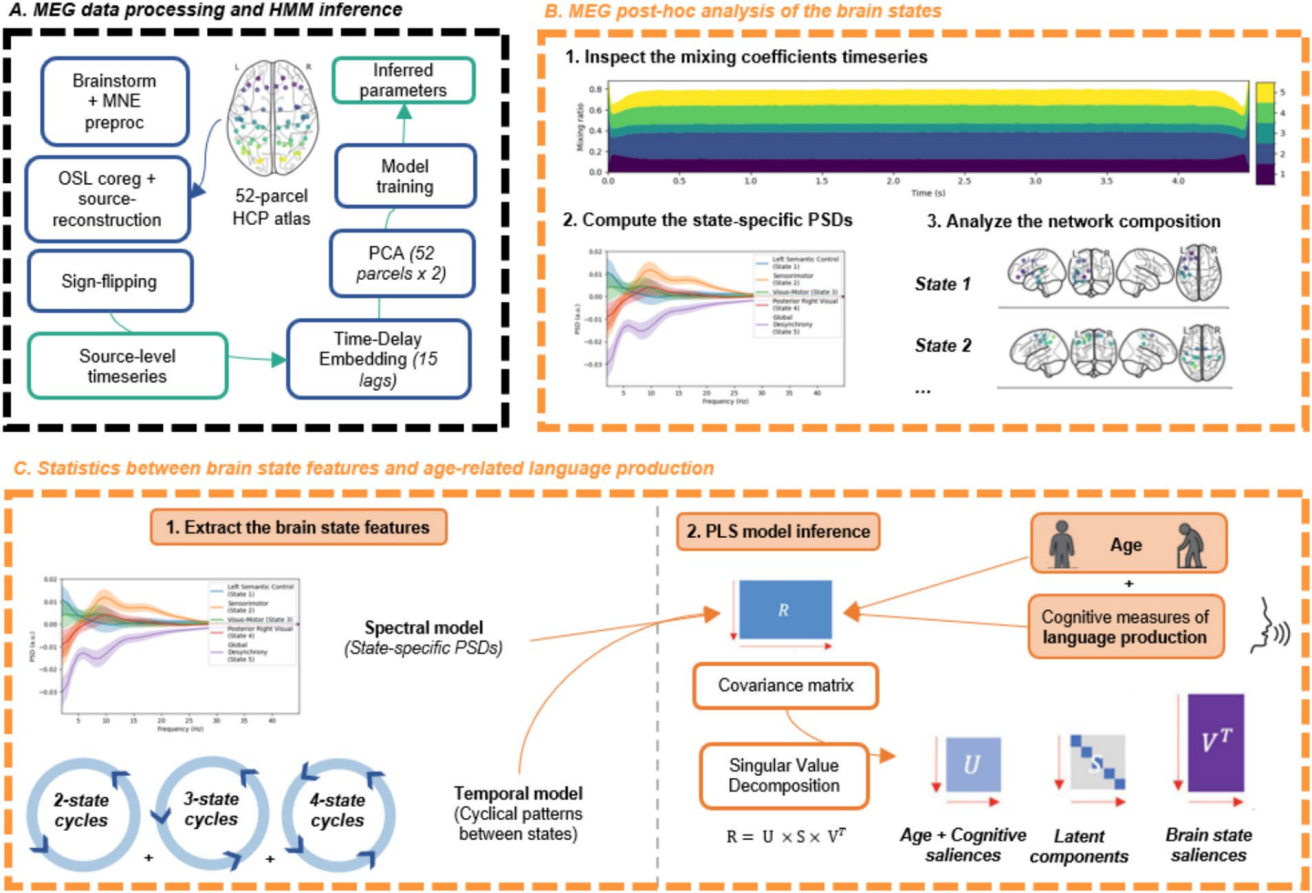
Schematic of the analysis pipeline. **(A)** is described in section 2.3 and 2.4; **(B)** in section 2.5; **(C)** in section 2.6.

*Preprocessing.* Preprocessing steps were initially carried out with the Brainstorm software,^1^ in MATLAB (Tadel et al., 2011). Temporal signal space separation (tSSS) (Taulu and Simola, 2006) was applied for noise reduction and offline head motion correction based on four head position indicators (HPIs). Data were notch filtered at 50 Hz and harmonics to remove power line noise. Further denoising was performed with Infomax ICA decomposing each block signal into 50 components before rejecting ECG/EOG-related artifacts (see Figure 2).

After the removal of bad trials (visualization), remaining good trials were further preprocessed with MNE python. First, we applied bandpass filtering using a 5th-order IIR Butterworth filter [0.5–125 Hz] and downsampled to 250 Hz. Second, bad channels were interpolated using spherical spline interpolation (Perrin et al., 1989) to keep data dimensions consistent across participants. Third, each preprocessed trial was cropped between *t* = 3.5 and *t* = 8 s, thereby omitting the first 500 ms of the task condition. This was done to isolate the core sentence generation phase for downstream analyses, while excluding early evoked visuo-sensory responses common across participants related to the display of the cue (Figure 1). Finally, all trials were concatenated along the time dimension to obtain one continuous trial sequence for each participant.

#### 2.3.2 Co-registration

T1-weighted MR images were acquired with Siemens Espree 1.5 T MR imager during a 30-min scan following the experimental procedure: TR = 14 ms, TE = 4.87 ms, FA = 25°, spatial resolution = 1 mm isometric, FOV = 256, number of slices = 176. MEG-T1 co-registration was conducted using digitized anatomical fiducial points (bilateral pre-auricular points, nasion, headshape) by extracting the scalp’s surfaces, inner skull, and brain with FSL Brain Extraction Tool (Smith, 2002). We used a 2-mm T1w MNI template for 3 participants (2 older, 1 younger) due to bad skull stripping and manually validated all co-registration outputs.

#### 2.3.3 Source reconstruction

Following OHBA Software Library (OSL) guidelines, preprocessed data were bandpass filtered [1–45 Hz], and sources were reconstructed onto an 8-mm isotropic dipole grid. This reconstruction was based on a single-shell lead-field model in MNI space and employed a linearly constrained minimum variance (LCMV) scalar beamformer (Van Veen et al., 1997; Woolrich et al., 2011). Voxels were parcellated into 52 regions derived from the HCP-MMP 1.0 atlas (Kohl et al., 2023) in line with previous team work on lexical production (Guichet et al., 2026). Symmetric multivariate leakage reduction was applied to mitigate source leakage (i.e., artefactual correlations between parcel time courses) (Colclough et al., 2015), and dipole sign ambiguity was resolved using a sign-flipping algorithm (Vidaurre et al., 2018).

### 2.4 MEG analysis: hidden Markov model

A Hidden Markov Model (HMM) was employed to model MEG activity. This model assumes that oscillatory activity can be generated from a sequence of distinct brain states whose activations vary over time. As recommended for task data, we selected the DyNeMo variant which allows states to overlap at each timepoint instead of being mutually exclusive, and explicitly accounts for long-range temporal dependencies.

For a detailed mathematical description of the observation model, please refer to Gohil et al. (2022). All modeling steps were carried out with the OHBA Software Library Dynamics Toolbox (*osl-dynamics*; Gohil et al., 2023).

#### 2.4.1 Data preparation

We prepared source-space data by adding time-lagged versions of each channel. These lags allowed the inference of spectral properties such as oscillatory amplitude and phase synchronization. As recommended, we used 15 lags evenly distributed around each timepoint (7 lags before and 7 lags after), which corresponds to a temporal window of ± 28 ms. Considering our 52 parcels, this introduced 728 (14 × 52) off-diagonal elements to the covariance matrix used for model training. To prevent overfitting, principal component analysis (PCA) reduced this space to twice the number of parcels (i.e., 104) before z-scoring the data across the time dimension.

#### 2.4.2 Model inference

Model hyperparameters were fine-tuned across the first runs based on training stability and convergence and subsequently set as follow: *sequence length* = 100, *batch size* = 16, *epochs* = 50, *learning rate* = 1e-3. We conducted 10 runs for *S* = 4, 6, or 8 states to find the optimal number of brain states fitting our data. We selected *S* = 6 states as the best compromise between model complexity and neurofunction interpretability based on the observed spatio-spectral features. As expected with our small sample size, we observed substantial variability across the 10 runs. Therefore, as recommended by Alonso and Vidaurre (2023), we employed hierarchical clustering to derive a more reliable solution that considers all runs rather than choosing the single best one. This procedure works in three steps: (i) we compute the Pearson correlation between every pair of state timeseries to build the distance matrix; (ii) we apply Ward’s hierarchical clustering algorithm to group similar states together across runs; (iii) the original state timeseries within each cluster is averaged to produce the set of states that occur consistently across runs.

### 2.5 MEG p*ost-hoc* analysis

#### 2.5.1 Power spectral density

Once trained, the model provides a timeseries of the mixing coefficients, indicating the mixing ratio between brain states at each time point (e.g., 20% State 1, 10% State 2, etc.). To extract the power spectrum density (PSD) of each state, we first computed the Welch spectrogram from source-space data and regressed it on the mixing coefficients using the GLM-spectrum approach (Quinn et al., 2024). The regression coefficients can then be interpreted as state-specific PSDs for each subject.

#### 2.5.2 Outliers

Initial inspection showed that State 4 resumed high-amplitude aperiodic activity across the whole-brain, whose parcel-averaged power was 3.62 z-units away the mean power observed in the other five states. This aperiodic profile was further confirmed by visualizing the subject-specific PSDs (see Supplementary Figures S1, S2, Supplementary Information). Relatedly, we excluded three outliers in the older age group which showed strong aperiodic activity in this state compared to the other subjects (see Supplementary Figure S1, Supplementary Information). This brings the final sample size to *N* = 7 in this age group. These exclusions were done prior to any statistical analysis linking the states to behavioral variables, avoiding any circular bias. Because the regression coefficients are estimated with respect to the mean of all states, we refitted the GLM-spectrum after excluding this aperiodic state to focus only on oscillatory activities. This brings the final number of states to *S* = 5.

#### 2.5.3 Network composition

To describe the states in terms of brain networks, we extended a method validated in previous teamwork (Guichet et al., 2026). First, we computed subject-specific power maps for each state by averaging each PSD across frequencies. Second, we computed the volumetric overlap between our 52 parcels and a 9-resting-state-network (RSN) atlas (Ji et al., 2019). The network composition of each state and subject was obtained by calculating the inner product between each subject’s power map (*dim:* 5 states x 52 parcels) and the volumetric overlap (*dim:* 52 parcels x 5 networks). We then averaged across subjects to obtain a single group-level network description whereby each state is associated with many resting-state networks (*dim*: 5 states x 9 networks). This approach allowed us to explicitly take into account the inter-individual variability in the states’ spatial layout.

### 2.6 Statistics

To examine how age and cognitive performances jointly modulate task dynamics, we employed Partial Least Squares correlation analysis^2^ in MATLAB R2025b. PLS is a multivariate technique that extracts latent components by maximizing the covariance between two datasets: brain features (*X matrix*) and a behavioral profile comprising age and cognitive scores (*Y matrix*). PLS offers a principled way to manage: (i) multicollinearity across features, (ii) contexts where there are more features than participants (Mihalik et al., 2022); and (iii) enhances the robustness of individual differences (DeYoung et al., 2025).

#### 2.6.1 PLS inference

The covariance matrix (R = X*Y^T^) was decomposed using singular value decomposition (SVD), resulting in R = USV^T^. Each latent component is defined by a singular value (s), representing the shared covariance, and a set of brain (u) and behavioral (v) saliences that denote the contribution of each feature to the component. Statistical significance of each component was assessed using 10,000 permutations, and *p*-values were adjusted at False Discovery Rate (FDR) across the components of each PLS model. Robustness of saliences was evaluated using 1,000 bootstrap resamples. We then calculated a Bootstrap Sampling Ratio (BSR) for each feature, defined as the observed salience weight divided by its bootstrapped standard deviation. Features with a BSR ± 3 were considered robust contributors, corresponding to approximately a 99% confidence interval (Krishnan et al., 2011).

#### 2.6.2 Behavioral matrix preparation

The behavioral matrix (Y) was constructed from performance scores on the TMT A/B, as well as Verbal and Semantic Fluency tests. TMT scores were inverted so that higher values consistently indicated better performance. To isolate the specific effect of age and cognition, we regressed several covariates: anxiety and depression levels (HADS), gender, total intracranial volume (TIV), MMSE, BREF score, and Mill-Hill vocabulary score. The resulting residuals were quantile-normalized to ensure Gaussianity. Finally, age was appended as a continuous variable and the entire matrix was z-scored across subjects.

#### 2.6.3 Brain feature matrix

This behavioral dataset was examined in relation to both spectral and temporal brain state properties through two PLS models:

- (*Model 1*) Spectral model: For each subject and state, we concatenated the PSD profile and z-scored across subjects. We limited our analysis to the top 10 parcels to ensure spectral relevance, that is focusing on each state’s highest group-average activity.
- (*Model 2*) Temporal model: We developed a method to capture cyclical patterns beyond simple pairwise state-to-state transitions. This approach groups brain states into higher-order recurrent sequential units (cycles of lengths 2, 3, and 4), enabling us to quantify how the brain state dynamics are segmented into recurring processing blocks during sentence production. Importantly, the cycles are estimated across all sentence generation periods combined, thus capturing the global organization of state sequences rather than the exact moment when each sequence occurs within a sentence.

First, we computed the inner product of the state time courses (*α*) to obtain the transition probability matrix, *T*. To focus exclusively on state-to-state dynamics, we zeroed the diagonal elements and then normalized each row to obtain a jump-transition matrix, *P* (Equation 1):


$$T_{\mathrm{ij}}=\sum_{t=1}^{N-1}\alpha_{t,i}\alpha_{t+1,j};P_{\mathrm{ij}}=\frac{T_{\mathrm{ij}}}{\sum l\neq i\;\;T_{\mathrm{il}}}\;\;\;$$ (1)

where *N* denotes the total number of timepoints in the session, and the denominator in the second expression represents the total flux out of state *i* into any other state *l*, excluding self-transitions.

This approach leverages the full posterior distribution of the HMM, modeling ‘soft’ transitions rather than the hard assignments typically used with binarized time courses, thereby respecting the model’s uncertainty. Considering the 5 states identified in this study (after exclusion of aperiodic activity), we generated a list of *M* = 60 unique cycles of lengths *k* ∈{2,3,4} . For each cycle ∁ , the probability ϕ(∁) is calculated as the geometric mean of the transition probabilities along the sequence of states $\langle s_{j},\ldots ,s_{k}\rangle$ (Equation 2):


$$\phi (∁)={\left(\prod_{j=1}^{k}P_{s_{j}s_{j}\;\mathrm{mod}\;k+1}\right)}^{1/k}\;\;\;$$ (2)

This formulation ensures the metric is normalized for cycle length and captures non-trivial dynamics as cycles containing ‘weak link’ transitions are penalized (if any link is near zero, the entire metric approaches zero). Because the resulting cycle values exist in a compositional space, we applied the centered-log-ratio (CLR) transform to linearize the data (Equation 3):


$$\mathrm{CLR}(\phi {\left(∁\right)}_{m})=\ln(\frac{\phi {\left(∁\right)}_{m}}{g(\phi )})$$ (3)

where g(ϕ) is the geometric mean of all computed cycles (Equation 4):


$$g(\phi )={\left(\prod_{m=1}^{M}\phi {\left(∁\right)}_{m}\right)}^{1/M}\;\;$$ (4)

This procedure was repeated for each subject and the resulting cycle values were z-scored across subjects in preparation for PLS.

## 3 Results

### 3.1 Description of the brain states during sentence generation

**Figure 3:**
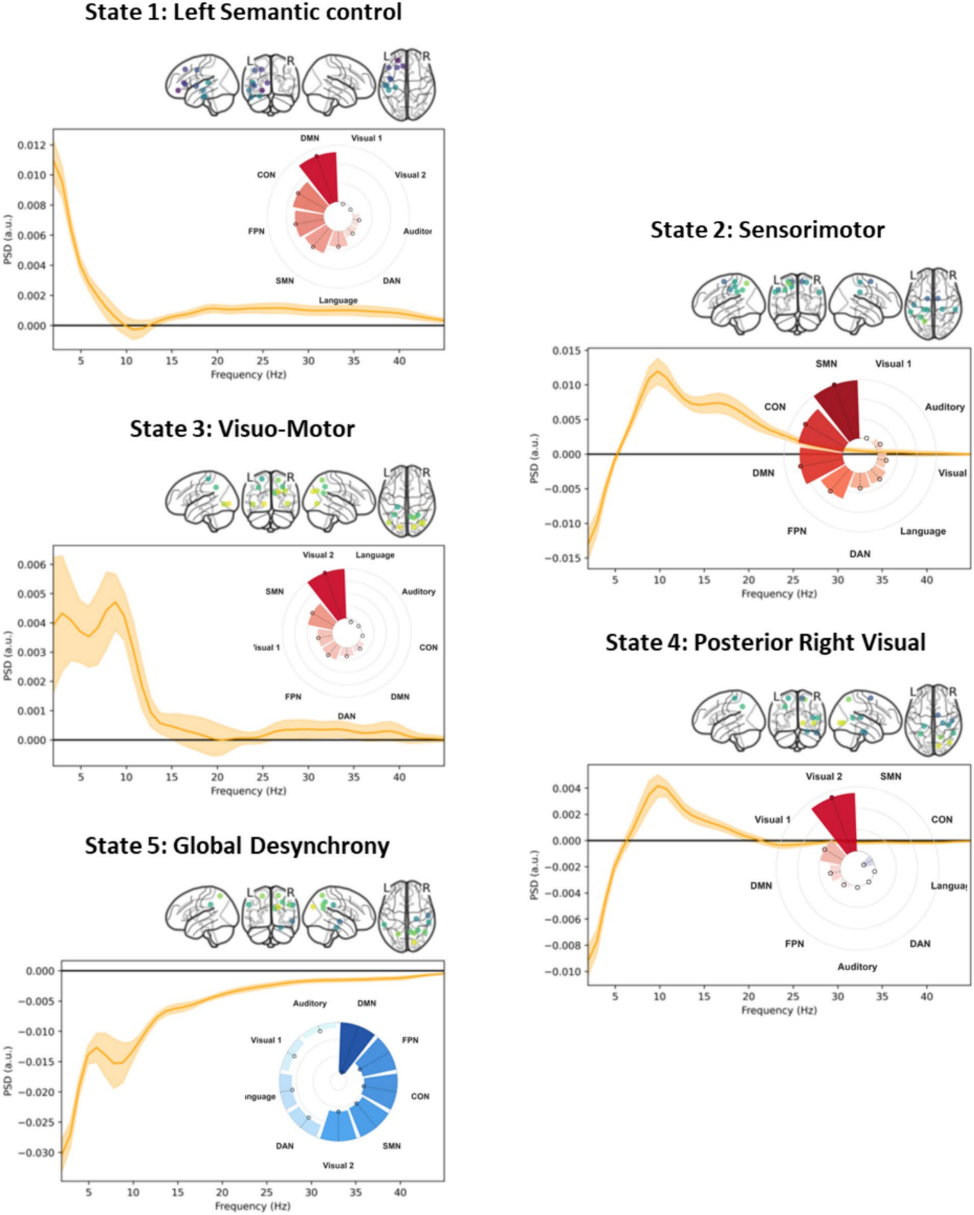
Dynamic spatio-spectral profiles of Sentence Generation. The main plot illustrates the group-average power spectra of each state across all 52 channels. Bandwidths represent the standard error across subjects. Brain plot. Illustrates each state’s 10 most-powered channels on average across all subjects. Radar plot. Illustrates the resting-state network topography of each state (red = synchrony, blue = desynchrony). *DMN (Default Mode), FPN (Fronto-Parietal), CON (Cingulo-opercular), DAN (Dorso-Attentional), SMN (Sensorimotor).*

The Hidden Markov Model revealed five recurrent brain states during the sentence generation period. Figure 3 illustrates the states’ network topography and spectral signature (see also Supplementary Figures S3, S4, Supplementary Information).

State 1 highlights overlaps between left-lateralized communication between the DMN and attentional systems (FPN and CON) in low frequencies (delta/theta bands < 8 Hz) and aperiodic activity (1/f slope), a regime typically associated with top-down cognitive control and lexico-semantic retrieval (Cavanagh et al., 2012; Nigbur et al., 2011; Zheng and Piai, 2025). State 2 engages similar overlaps with the DMN and attentional systems (FPN and CON), but with the SMN as the main driver in a broad alpha-beta band (10–30 Hz).

States 3 and 4 are visuo-posterior states dominated by alpha activity with clear peaks in the 8–12 Hz band. State 3 specifically binds with the SMN with an additional peak in the delta range whereas State 4 binds with the DMN. Finally, State 5 represents a period of right-dominant desynchronization with respect to the mean task activation, which appears as the counterpart of DMN synchronization in State 1.

**Figure 4:**
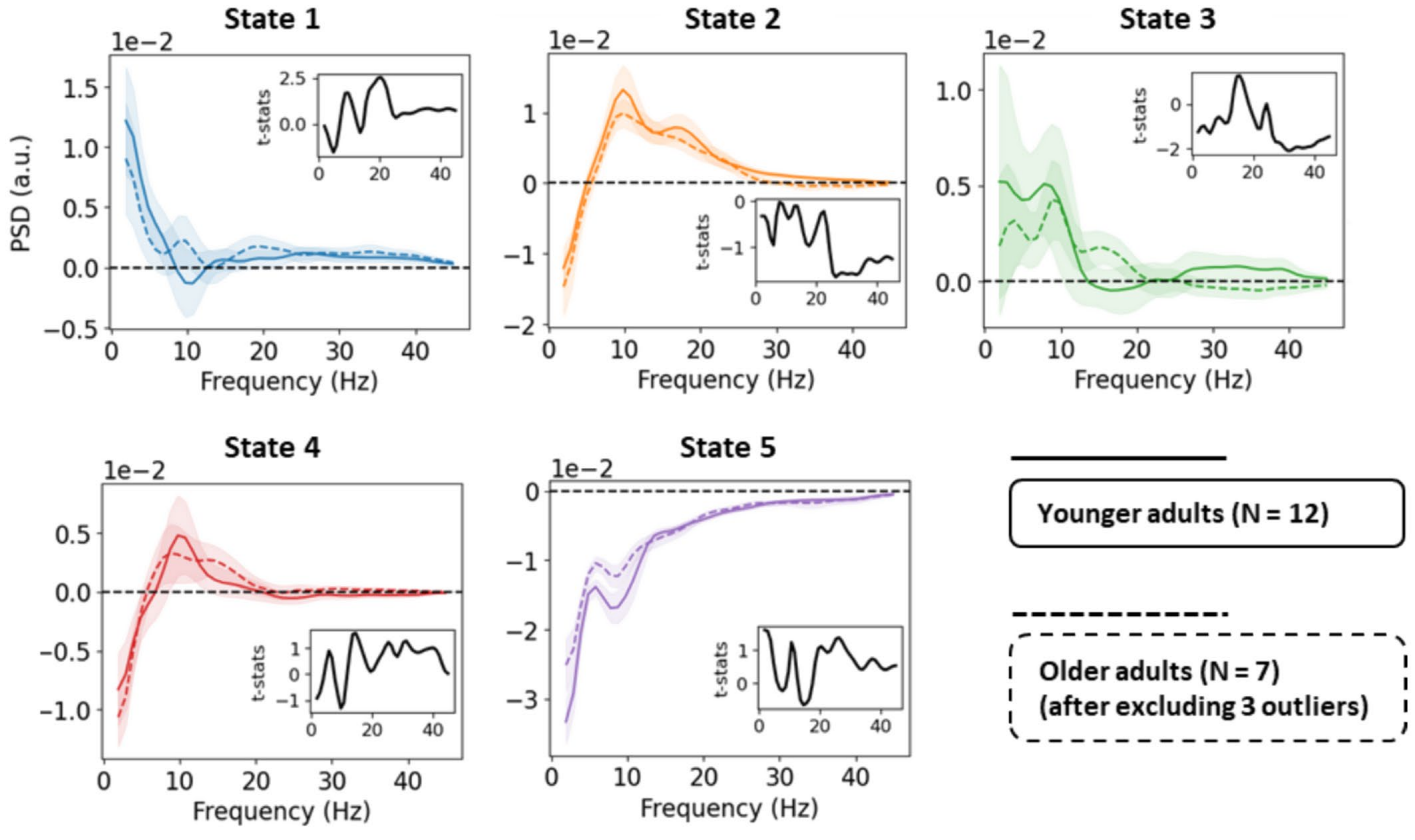
Cluster-based permutation results between age-group spectra. For each state, the plain line depicts the average spectrum of younger adults, and the dotted one that of older adults. The in-plot reports the t-statistic from 5,000-permutations. No significant clusters were identified following Bonferroni correction across states.

Initial cluster-permutation tests revealed no significant differences between age groups in neither of the five states (Figure 4). In the next section, we employed a latent modeling approach to capture more holistic patterns of change across the state space, considering the contribution of all states simultaneously.

### 3.2 Latent modeling between brain features, age, and cognitive performances

Together, the five states identified above constitute the basic dynamic units of the sentence generation task. Using the Partial Least Squares (PLS) analysis, we examined how their spectral properties and temporal organization covary with age and behavioral performances.

The PLS models identified a single significant latent component (*p_spectral_* = 0.016 and *p_temporal_* = 0.015), explaining, respectively, 39.5 and 50% of the total shared covariance. While neither component survived FDR correction (*pFDR_spectral_* = 0.079 and *pFDR_temporal_* = 0.075), spectral and temporal models converged on a similar behavioral profile. We begin by describing this behavioral profile, and then report the associated changes in oscillatory properties and sequential brain state organization.

#### 3.2.1 Behavioral profile

**Figure 5:**
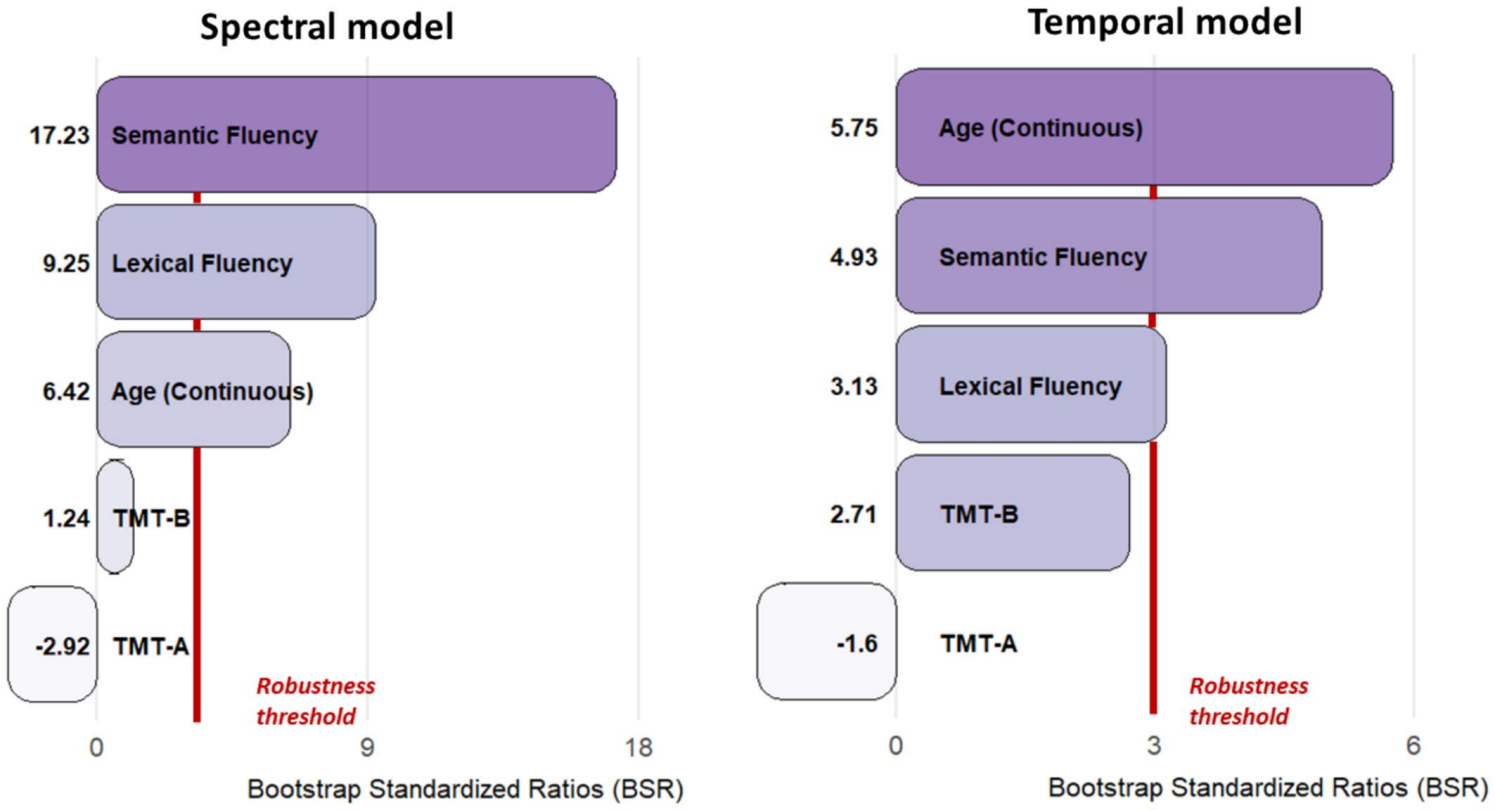
Salient demographic and behavioral features for each PLS model.

Overall, results suggest a coordinated time-frequency reorganization of sentence generation linked to age-related verbal fluency performances (Figure 5). Spectral features most robustly covaried with semantic and verbal fluency (BSR*_spectral_* = 17.23 and 9.25), whereas the temporal dynamics between states more robustly captured the effects of brain aging (BSR*_temporal_* = 5.75).

Although not robust contributors (|BSR| < 3), we observed a positive trend for cognitive flexibility (TMT B), especially covarying with the brain state temporal organization (BSR*_temporal_* = 2.71). This lack of robustness could be attributed to high variability across subjects as evidenced by a high bootstrap standard deviation (0.2), twice to the average of other behavioral measures in the temporal model (~ 0.1). By contrast, a negative trend was also observed for baseline processing speed (TMT A), especially covarying with spectral dynamics (BSR*_spectral_* = −2.92).

#### 3.2.2 Spectral model

**Figure 6:**
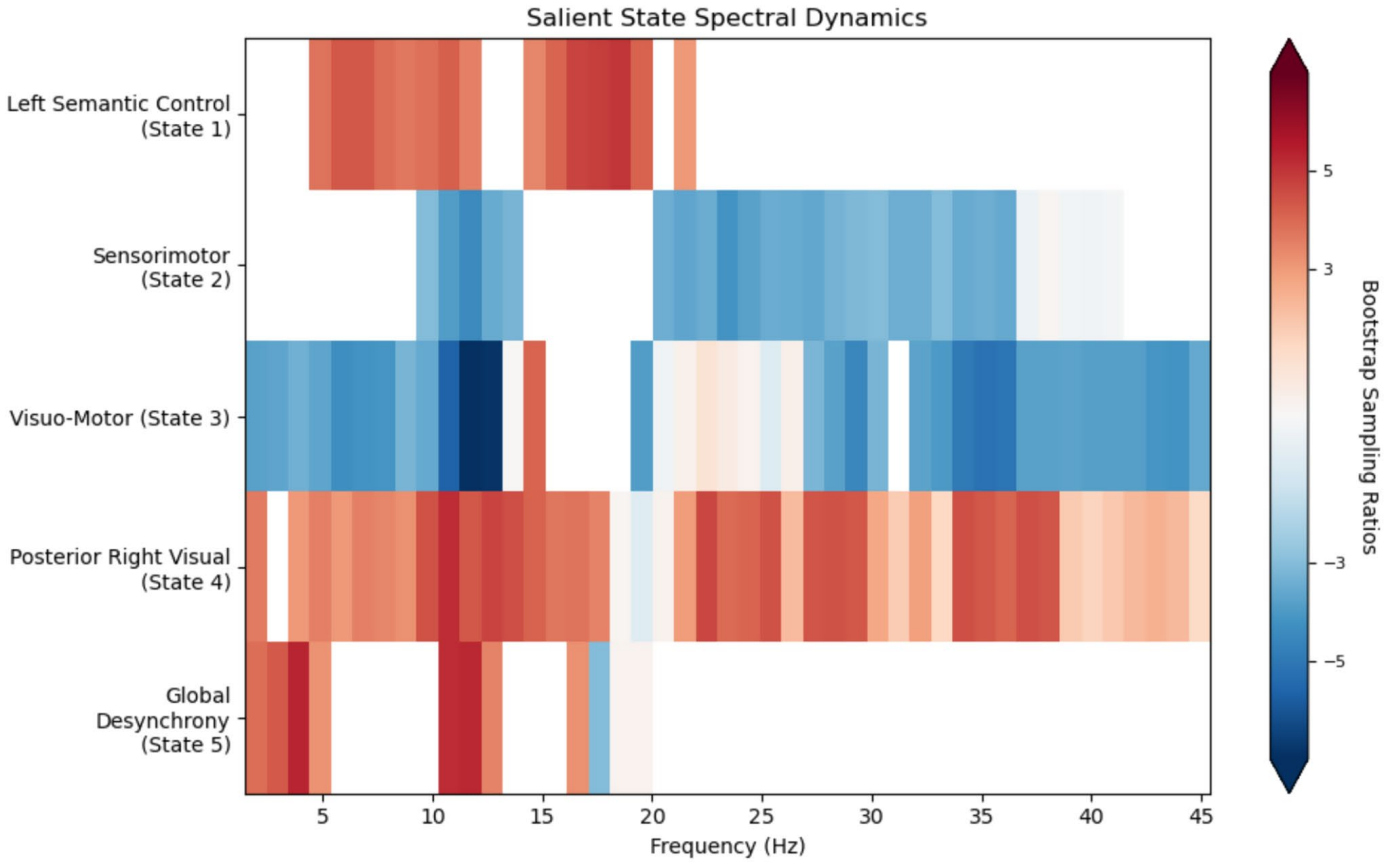
Salient spectral dynamics across brain states. A high BSR (red) indicates robust contribution to the latent brain variable, covarying with age and the behavioral profile shown in Figure 5.

Age and the associated behavioral profile (Figure 5) covaried with a large-scale redistribution of oscillatory power across states (Figure 6), particularly from the sensorimotor (State 2) and visuo-motor configurations (State 3) toward the states which bind with the higher-level DMN (State 1: semantic control and State 4: posterior right visual). This redistribution involved nearly the entire spectrum, although more robustly impacting the alpha (10–12 Hz), beta and low-gamma (20–37 Hz) bands. Additionally, we noted a less powerful desynchrony in State 5 (4 Hz peak and alpha 10–12 Hz band).

#### 3.2.3 Temporal model

**Figure 7:**
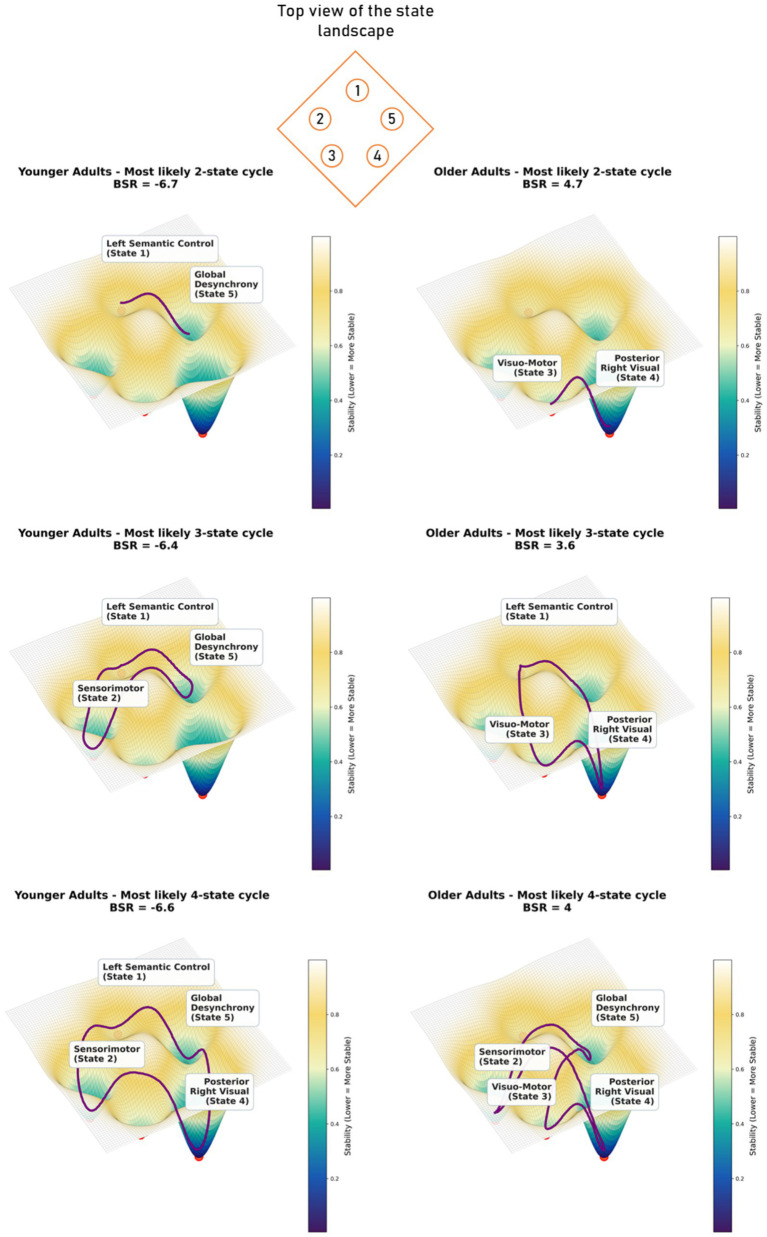
Most salient brain state cycles. Older adults’ cycles are associated with a positive BSR because they are positively correlated to the latent component shown in Figure 5; and conversely for younger adults’. Energy landscape. To visualize changes in cycle dynamics, we constructed a 3D energy landscape where states are organized in a circular layout (see Supplementary Information). The resulting landscape exhibits deep basins for states with high stability, and shallow valleys for more instable, transient states. Projected cycle dynamics. Salient brain state cycles are projected onto this landscape as smooth purple trajectories. BSR, Bootstrap Sampling Ratio.

The temporal PLS model provided complementary insights to the broad spectral changes identified previously, notably revealing changes in the processing stages of sentence generation (Figure 7).

While younger adults engaged in direct cycles involving the Left Semantic Control, Sensorimotor, and Desynchrony configurations (States 1, 2, 5), older adults exhibited a more segmented repertoire, additionally cycling through the Visuo-motor and Posterior Right Visual states (States 3 and 4). This suggests a form of temporal “segmentation” of the sentence generation stages in older adults, which covaried with better verbal fluency.

## 4 Discussion

This study investigated the neurophysiological mechanisms supporting naturalistic sentence generation in younger and older adults. Using Hidden Markov Modeling, we identified five co-active brain states during covert speech and demonstrated that their spatiotemporal properties are associated with age and verbal fluency. Taken together, our findings support our initial hypotheses. Healthy language aging appears to be characterized by an embodied semantic strategy, scaffolded by a coordinated time-frequency reorganization of large-scale brain dynamics spanning language-semantic, language-control, sensorimotor, and visual domains.

### 4.1 Segmented processing: an age-related compensatory mechanism?

Our analysis of state-transition cycles complements recent evidence that large-scale brain networks organize into structured, nontrivial temporal sequences during cognitive tasks (Van Es et al., 2025). As illustrated in Figure 7, we observed a marked shift in cycle dynamics with age. Younger adults predominantly relied on a direct loop involving left-lateralized semantic control (State 1), sensorimotor processing (State 2), and global desynchronization (State 5) during sentence generation. Together, these states seem to support controlled word retrieval and articulatory-motor preparation. In contrast, older adults exhibited a segmented dynamic in which this loop was split into two sub-cycles, each interfacing with additional visuo-posterior configurations (States 3 and 4). These states appear to act as intermediary perceptual integration step between articulatory-motor processing (State 3: Visual-SMN) and higher-level semantic retrieval (State 4: Visual-DMN). This finding suggests that maintaining verbal fluency in older adulthood may require traversing a more fragmented functional trajectory that supports motor imagery (State 3) and visually guided lexical access (State 4) (Roos et al., 2023).

This contribution of visuo-posterior activity to sentence generation in aging provides novel task-based MEG evidence that older adults adopt an “embodied semantic strategy” for language production, in support of the SENECA model (Guichet et al., 2026). It also aligns with the broader notion of predictive processing whereby multisensory inputs, here primarily visual, prime semantic representations to support complex cognition in aging (Brown et al., 2022; Ozkalp-Poincloux et al., 2026; Spreng and Turner, 2021).

From a metabolic standpoint, this predictive route can be interpreted as form of energy-efficient “temporal coding” that could emerge as “natural consequence of resource optimization” in older adulthood (Elias, 1955; Price and Gavornik, 2022). Since the aging brain faces a reduction in metabolic resources (Asimakidou et al., 2025), shifting from a streamlined three-state cycle to a segmented five-state dynamic is consistent with a compensatory mechanism that reduces signaling costs during language production (Hechler et al., 2023). In this framework, our results suggest that visuo-posterior integration could act as an efficient mediator between distinct processing segments.

Although speculative, this interpretation aligns with evidence indicating that older adults primarily allocate metabolic resources to posterior hub regions (Deery et al., 2024). Prior resting-state MEG findings have also highlighted dorso-posterior brain states as candidate for information integration in age-related language production (Guichet et al., 2026).

### 4.2 Spectral mechanisms of the semantic strategy

The brain states’ oscillatory dynamics were also associated with the age-related semantic strategy. We primarily observed a dissociation between states primarily involved in sensorimotor-related processing (State 2: SMN; State 3: Visual-SMN) and those associated with semantic-related networks (State 1: semantic control; State 4: Visual-DMN) (Figure 6). These state categories exhibited reciprocal spectral modulations with age: sensorimotor-related states exhibited broad suppression, whereas semantic configurations displayed robust power increases across frequencies.

One possible explanation is that, as the temporal sequence between semantic and sensorimotor processing becomes less efficient with age (e.g., slower processing speed), the brain redistributes its resources to support increased engagement from DMN-semantic hubs. This interpretation is compatible with the LARA-C model (Baciu and Roger, 2024) which emphasizes the recruitment of compensatory semantic pathways to support language production in aging, and suggests that older adults resolve lexical competition at the semantic level to mitigate control deficits (Zheng and Piai, 2025).

While such control deficits were not robustly identified due to our low sample size (trending associations with TMT performance), we still observed reduced theta-alpha desynchronization in older adults (State 5). This oscillatory dynamic aligns with recent evidence that alpha temporally coordinates theta processes (Beste et al., 2023), and could impact top-down control, attentional gating mechanisms (Foxe and Snyder, 2011; Pscherer et al., 2026), or perception-action integration (Eggert et al., 2025) in aging.

Nonetheless, it is important to note that the multivariate nature of PLS analysis limits the ability to isolate the independent contribution of specific frequency bands. Specifically, we did not find an expected association between beta power decreases and age-related semantic integration (Piai et al., 2014, 2015, 2020), but we observed enhanced alpha-band modulations in visuo-posterior states which could be interpreted as increased demands for semantic control in older adults (Guichet et al., 2026; Klimesch, 2012; Zioga et al., 2024). In sum, observed oscillatory changes suggest that age-related sentence production involves a coordinated, multi-spectral reorganization rather than the involvement of specific frequency bands.

### 4.3 Limitations and future directions

A primary limitation of this study is the relatively small sample size, particularly after exclusion of outliers in the older age group, which reduces statistical power and limits generalization. This constraint may partly explain the absence of more subtle effects with certain executive measures, such as TMT performance. In addition, the present covert sentence generation task may impose relatively low cognitive demands, which may have minimized larger age-group differences. Prior studies have shown that syntactic processing can remain preserved in older adults’ sentence production under low linguistic demands (Davidson et al., 2003; Hardy et al., 2022; Lee et al., 2022). Thus, future research will be necessary to validate the proposed segmentation mechanism in aging in larger cohorts, using more demanding linguistic paradigms that tap into working memory, such as complex lexico-syntactic constructions (Agmon et al., 2023; Sung et al., 2024), or by eliciting sentences with abstract concepts to control for the degree of embodied semantic representation (Diveica et al., 2025; Pexman et al., 2023). On that note, the sentence generated were not recorded in this study because of the covert speech paradigm. Future experiments could perform further semantic analysis to examine whether older adults use more action-oriented lemmas, which would support the hypothesis of embodied semantics for complex cognition in aging (Guichet et al., 2026).

Although MEG provides excellent temporal resolution, signal-to-noise ratio limitations were evident, with the HMM framework isolating a low-frequency state likely reflecting noise. Moreover, future work could explicitly model for the aperiodic component of the signal, which is known to flatten with age (Mooraj et al., 2025), especially with external task engagement (Kosciessa et al., 2024; Podvalny et al., 2015). Beyond cortical dynamics, cortico-cerebellar loops and hippocampal contributions are increasingly recognized as central to predictive updating of internal models, especially during language processing (Casto et al., 2026; Yaghoubi et al., 2026). Integrating these subcortical sources, for example through MEG-fMRI approaches or invasive recordings such as stereo-EEG, will be essential to further elucidate the high-gamma band processing of embodied semantic representations in the aging brain (Dupont et al., 2025).

## 5 Conclusion

This study investigated the MEG brain state dynamics supporting sentence generation in healthy aging using a cohort of 22 French participants. Overall, results suggest that semantic processing constitutes a compensatory adaptation for language production in aging, supported by a spatially distributed, spectrally diverse, and temporally segmented brain architecture.

This architecture is compatible with a compensatory mechanism that segments processing between lexico-semantic retrieval and articulatory-motor preparation in older adulthood. These processing stages may be interfaced by intermediary visuo-posterior activity, which could serve as a functional bridge within the processing sequence. Finally, we speculate that such an organization may contribute to a relatively resource-efficient functional layout in aging, helping to maintain language fluency despite a reduced metabolic budget.

## Data Availability

The raw data supporting the conclusions of this article will be made available by the authors, without undue reservation.

## Footnotes

1
http://neuroimage.usc.edu/brainstorm

2
https://github.com/MIPLabCH/myPLS

## References

[ref1] Agmon G. Pradhan S. Ash S. Nevler N. Liberman M. Grossman M. . (2023). Automated measures of syntactic complexity in natural speech production: older and younger adults as a case study. PsychArchives. doi: 10.23668/PSYCHARCHIVES.13145 PMC12510242 38215342

[ref2] Aliko S. Wang B. Small S. L. Skipper J. I. (2023). The entire brain, more or less, is at work: ‘language regions’ are artefacts of averaging [preprint]. Neuroscience. doi: 10.1101/2023.09.01.555886

[ref3] Alonso S. Vidaurre D. (2023). Toward stability of dynamic FC estimates in neuroimaging and electrophysiology: solutions and limits. Netw. Neurosci. 7, 1389–1403. doi: 10.1162/netn_a_00331, 38144684 PMC10713011

[ref4] Antzoulatos E. G. Miller E. K. (2016). Synchronous beta rhythms of frontoparietal networks support only behaviorally relevant representations. eLife 5:e17822. doi: 10.7554/eLife.17822, 27841747 PMC5148609

[ref5] Asimakidou E. Pluchino S. Silva B. A. Peruzzotti-Jametti L. (2025). The metabolic engine of cognition: microglia–neuron interactions in health, ageing and disease. Nat. Metab. 7, 2395–2413. doi: 10.1038/s42255-025-01409-4, 41272201

[ref6] Baciu M. Banjac S. Roger E. Haldin C. Perrone-Bertolotti M. Lœvenbruck H. . (2021). Strategies and cognitive reserve to preserve lexical production in aging. GeroScience 43, 1725–1765. doi: 10.1007/s11357-021-00367-5, 33970414 PMC8492841

[ref7] Baciu M. Boudiaf N. Cousin E. Perrone-Bertolotti M. Pichat C. Fournet N. . (2016). Functional MRI evidence for the decline of word retrieval and generation during normal aging. Age 38:3. doi: 10.1007/s11357-015-9857-y, 26711670 PMC5005885

[ref8] BaciuM. RogerE. (2024). Finding the words: how does the aging brain process language? A focused review of brain connectivity and compensatory pathways. Top. Cogn. Sci.:12736. doi: 10.1111/tops.1273638734967

[ref9] Badre D. Wagner A. D. (2007). Left ventrolateral prefrontal cortex and the cognitive control of memory. Neuropsychologia 45, 2883–2901. doi: 10.1016/j.neuropsychologia.2007.06.015, 17675110

[ref10] Baltes P. B. Lindenberger U. (1997). Emergence of a powerful connection between sensory and cognitive functions across the adult life span: a new window to the study of cognitive aging? Psychol. Aging 12, 12–21. doi: 10.1037/0882-7974.12.1.12, 9100264

[ref11] Banjac S. Roger E. Cousin E. Perrone-Bertolotti M. Haldin C. Pichat C. . (2021). Interactive mapping of language and memory with the GE2REC protocol. Brain Imaging Behav. 15, 1562–1579. doi: 10.1007/s11682-020-00355-x, 32761343 PMC8286228

[ref12] Bannon J. Ferreira V. S. Stasenko A. Gollan T. H. (2024). Competition accumulates in successive retrieval of proper names. Mem. Cogn. 52, 197–210. doi: 10.3758/s13421-023-01455-x, 37721701

[ref13] Bastos A. M. Usrey W. M. Adams R. A. Mangun G. R. Fries P. Friston K. J. (2012). Canonical microcircuits for predictive coding. Neuron 76, 695–711. doi: 10.1016/j.neuron.2012.10.038, 23177956 PMC3777738

[ref14] Bayram M. Palluel-Germain R. Lebon F. Durand E. Harquel S. Perrone-Bertolotti M. (2023). Motor imagery training to improve language processing: what are the arguments? Front. Hum. Neurosci. 17:982849. doi: 10.3389/fnhum.2023.982849, 36816506 PMC9929469

[ref15] Benítez-Burraco A. Ivanova O. (2023). Revisiting the hypothesis of language retrogenesis from an evolutionary perspective. Neuropsychology 37, 501–518. doi: 10.1037/neu0000886, 36729501

[ref16] Benítez-Burraco A. Ivanova O. (2024). Language in healthy and pathological ageing: methodological milestones and challenges. Int. J. Lang. Commun. Disord. 59, 4–12. doi: 10.1111/1460-6984.13003, 38149881 PMC13507593

[ref17] Beste C. Münchau A. Frings C. (2023). Towards a systematization of brain oscillatory activity in actions. Commun. Biol. 6:137. doi: 10.1038/s42003-023-04531-9, 36732548 PMC9894929

[ref18] Blanco N. J. Love B. C. Ramscar M. Otto A. R. Smayda K. Maddox W. T. (2016). Exploratory decision-making as a function of lifelong experience, not cognitive decline. J. Exp. Psychol. Gen. 145, 284–297. doi: 10.1037/xge0000133, 26726916 PMC4755819

[ref19] Borne A. Lemaitre C. Bulteau C. Baciu M. Perrone-Bertolotti M. (2024). Unveiling the cognitive network organization through cognitive performance. Sci. Rep. 14:11645. doi: 10.1038/s41598-024-62234-5, 38773246 PMC11109237

[ref20] Bornkessel-Schlesewsky I. Schlesewsky M. (2019). Toward a neurobiologically plausible model of language-related, negative event-related potentials. Front. Psychol. 10:298. doi: 10.3389/fpsyg.2019.00298, 30846950 PMC6393377

[ref21] Boudiaf N. Laboissière R. Cousin É. Fournet N. Krainik A. Baciu M. (2018). Behavioral evidence for a differential modulation of semantic processing and lexical production by aging: a full linear mixed-effects modeling approach. Aging Neuropsychol. Cognit. 25, 1–22. doi: 10.1080/13825585.2016.1257100, 27883290

[ref22] Bourguignon N. Lo Bue S. (2025). An emergentist account of language in the brain—seeking neural synergies behind human uniqueness. J. Cogn. Neurosci. 37, 1717–1734. doi: 10.1162/jocn_a_02331 40198105

[ref23] Brown R. M. Gruijters S. L. K. Kotz S. A. (2022). Prediction in the aging brain: merging cognitive, neurological, and evolutionary perspectives. J. Gerontol. 77, 1580–1591. doi: 10.1093/geronb/gbac062, 35429160 PMC9434449

[ref24] Brown R. McNeill D. (1966). The “tip of the tongue” phenomenon. J. Verb. Learn. Verb. Behav. 5, 325–337. doi: 10.1016/S0022-5371(66)80040-3

[ref26] Cardebat D. Doyon B. Puel M. Goulet P. Joanette Y. (1990). Formal and semantic lexical evocation in normal subjects. Performance and dynamics of production as a function of sex, age and educational level. Acta Neurol. Belg. 90, 207–217. 2124031

[ref27] Casto C. Poliak M. Tuckute G. Small H. Sherlock P. Wolna A. . (2026). The cerebellar components of the human language network. Neuron 114, 1504–1523.e11. doi: 10.1016/j.neuron.2025.12.030, 41576956 PMC13551672

[ref28] Cavanagh J. F. Zambrano-Vazquez L. Allen J. J. B. (2012). Theta lingua franca: a common mid-frontal substrate for action monitoring processes. Psychophysiology 49, 220–238. doi: 10.1111/j.1469-8986.2011.01293.x, 22091878 PMC3262926

[ref29] Chao Z. C. Huang Y. T. Wu C.-T. (2022). A quantitative model reveals a frequency ordering of prediction and prediction-error signals in the human brain. Commun. Biol. 5:1076. doi: 10.1038/s42003-022-04049-6, 36216885 PMC9550773

[ref30] Colclough G. L. Brookes M. J. Smith S. M. Woolrich M. W. (2015). A symmetric multivariate leakage correction for MEG connectomes. NeuroImage 117, 439–448. doi: 10.1016/j.neuroimage.2015.03.071, 25862259 PMC4528074

[ref31] Cui G. Ren Y. Zhou X. (2025). Language as a modulator to cognitive and neurological systems. Acta Psychol. 254:104803. doi: 10.1016/j.actpsy.2025.104803, 39965507

[ref32] Cutler R. A. Mirjalili S. Pham P. Devulapalli H. Zafar S. Duarte A. (2025). Semantic memory space becomes denser with age. Neuropsychologia 208:109083. doi: 10.1016/j.neuropsychologia.2025.109083, 39863135

[ref33] Dartinet V. Martinaud O. (2005). La BREF, une batterie rapide d’évaluation frontale. NPG Neurologie - Psychiatrie - Gériatrie 5, 43–46. doi: 10.1016/S1627-4830(05)82606-6

[ref34] Davidson D. J. Zacks R. T. Ferreira F. (2003). Age preservation of the syntactic processor in production. J. Psycholinguist. Res. 32, 541–566. doi: 10.1023/A:1025402517111, 14564992 PMC1751477

[ref35] Deery H. A. Liang E. X. Siddiqui M. N. Murray G. Voigt K. Di Paolo R. . (2024). Reconfiguration of metabolic connectivity in ageing. Commun. Biol. 7:1600. doi: 10.1038/s42003-024-07223-0, 39616242 PMC11608353

[ref36] Deltour J. J. (1993). Echelle de vocabulaire Mill Hill de J. C. Raven: Adaptation française et normes comparées du Mill Hill et du Standard Progressive Matrices (PM38). Manuel et Annexes.

[ref001] Deloche G. Hannequin D. (1997). Test de dénomination orale d’images: DO 80. Available online at: https://www.sudoc.fr/183587022, 39616242

[ref37] DeYoung C. G. Hilger K. Hanson J. L. Abend R. Allen T. A. Beaty R. E. . (2025). Beyond increasing sample sizes: optimizing effect sizes in neuroimaging research on individual differences. J. Cogn. Neurosci. 37, 1023–1034. doi: 10.1162/jocn_a_02297, 39792657

[ref38] Diveica V. Muraki E. J. Binney R. J. Pexman P. M. (2025). Contrasting the organization of concrete and abstract word meanings. Psychon. Bull. Rev. 32, 1814–1826. doi: 10.3758/s13423-025-02671-z, 40032746

[ref39] Dupont W. Dornier V. Palluel-Germain R. Robin A. Lachaux J.-P. Kahane P. . (2025). Reading about sensations recruits the posterior insula: an intracranial EEG study. iScience 28:113874. doi: 10.1016/j.isci.2025.113874, 41312377 PMC12648984

[ref40] Eggert E. Prochnow A. Talebi N. Frings C. Münchau A. Beste C. (2025). Uncovering the role of directed connectivity in alpha and theta band activity for sustaining perception-action links. Commun. Biol. 8:1147. doi: 10.1038/s42003-025-08601-y, 40753307 PMC12317987

[ref41] Elias P. (1955). Predictive coding—I. IEEE Trans. Inf. Theory 1, 16–24. doi: 10.1109/TIT.1955.1055126

[ref42] Endress A. D. (2019). Duplications and domain-generality. Psychol. Bull. 145, 1154–1175. doi: 10.1037/bul0000213, 31670549

[ref43] Facal D. Juncos-Rabadán O. Rodríguez M. S. Pereiro A. X. (2012). Tip-of-the-tongue in aging: influence of vocabulary, working memory and processing speed. Aging Clin. Exp. Res. 24, 647–656. doi: 10.1007/BF03654837, 22960259

[ref44] Fargier R. Laganaro M. (2023). Referential and inferential production across the lifespan: different patterns and different predictive cognitive factors. Front. Psychol. 14:1237523. doi: 10.3389/fpsyg.2023.1237523, 38022984 PMC10643179

[ref45] Federmeier K. D. Jongman S. R. Szewczyk J. M. (2020). Examining the role of general cognitive skills in language processing: a window into complex cognition. Curr. Dir. Psychol. Sci. 29, 575–582. doi: 10.1177/0963721420964095, 33584021 PMC7877800

[ref46] Forkel S. J. Hagoort P. (2024). Redefining language networks: connectivity beyond localised regions. Brain Struct. Funct. 229, 2073–2078. doi: 10.1007/s00429-024-02859-4, 39551912 PMC11611971

[ref47] Foxe J. J. Snyder A. C. (2011). The role of alpha-band brain oscillations as a sensory suppression mechanism during selective attention. Front. Psychol. 2:154. doi: 10.3389/fpsyg.2011.00154, 21779269 PMC3132683

[ref48] Fujioka T. Trainor L. J. Large E. W. Ross B. (2009). Beta and gamma rhythms in human auditory cortex during musical beat processing. Ann. N. Y. Acad. Sci. 1169, 89–92. doi: 10.1111/j.1749-6632.2009.04779.x 19673759

[ref49] Gilis S. Lefebvre L. Simoes Loureiro I. (2025). Effects of semantic interference and facilitation on lexical retrieval: evolution in healthy aging. Exp. Aging Res. 52, 331–351. doi: 10.1080/0361073X.2025.2585769, 41204422

[ref50] Gohil C. Huang R. Roberts E. Van Es M. W. J. Quinn A. J. Vidaurre D. . (2023). Osl-dynamics: a toolbox for modelling fast dynamic brain activity. eLife. doi: 10.7554/eLife.91949.2 PMC10945565 38285016

[ref51] Gohil C. Roberts E. Timms R. Skates A. Higgins C. Quinn A. . (2022). Mixtures of large-scale dynamic functional brain network modes. NeuroImage 263:119595. doi: 10.1016/j.neuroimage.2022.119595, 36041643 PMC7618940

[ref52] Gollan T. H. Goldrick M. (2019). Aging deficits in naturalistic speech production and monitoring revealed through reading aloud. Psychol. Aging 34, 25–42. doi: 10.1037/pag0000296, 30265018 PMC6367048

[ref53] Guichet C. Achard S. Mermillod M. Baciu M. (2026). Cognitive flexibility and brain network energy in healthy aging: an Allostatic perspective from the SENECA model. Imaging Neurosci. 4:IMAG.a.1091. doi: 10.1162/IMAG.a.1091, 41537050 PMC12797148

[ref54] Guichet C. Banjac S. Achard S. Mermillod M. Baciu M. (2024). Modeling the neurocognitive dynamics of language across the lifespan. Hum. Brain Mapp. 45:e26650. doi: 10.1002/hbm.26650, 38553863 PMC10980845

[ref55] Guichet C. Harquel S. Achard S. Mermillod M. Baciu M. (2026). Lifespan oscillatory dynamics in lexical production: a population-based MEG resting-state analysis. Imaging Neurosci. 3:551. doi: 10.1162/imag_a_00551, 40800836 PMC12319834

[ref56] Hagoort P. (2019). The neurobiology of language beyond single-word processing. Science 366, 55–58. doi: 10.1126/science.aax0289, 31604301

[ref57] Hagoort P. (2023). The language marker hypothesis. Cognition 230:105252. doi: 10.1016/j.cognition.2022.105252, 36115201

[ref58] Hanslmayr S. Spitzer B. Bauml K.-H. (2009). Brain oscillations dissociate between semantic and nonsemantic encoding of episodic memories. Cereb. Cortex 19, 1631–1640. doi: 10.1093/cercor/bhn197, 19001457

[ref59] Hardy S. M. Segaert K. Wheeldon L. (2022). Age-related effects on lexical, but not syntactic, processes during sentence production. Lang. Cogn. Neurosci. 37, 120–134. doi: 10.1080/23273798.2021.1948081

[ref60] Hechler A. De Lange F. P. Riedl V. (2023). The energy metabolic footprint of predictive processing in the human brain. Neuroscience. doi: 10.1101/2023.12.08.570804

[ref62] Hoffman P. Morcom A. M. (2018). Age-related changes in the neural networks supporting semantic cognition: a meta-analysis of 47 functional neuroimaging studies. Neurosci. Biobehav. Rev. 84, 134–150. doi: 10.1016/j.neubiorev.2017.11.010 29183684

[ref63] Hoyau E. Roux-Sibilon A. Boudiaf N. Pichat C. Cousin E. Krainik A. . (2018). Aging modulates fronto-temporal cortical interactions during lexical production. A dynamic causal modeling study. Brain Lang. 184, 11–19. doi: 10.1016/j.bandl.2018.06.003, 29913316

[ref64] Jensen O. Gips B. Bergmann T. O. Bonnefond M. (2014). Temporal coding organized by coupled alpha and gamma oscillations prioritize visual processing. Trends Neurosci. 37, 357–369. doi: 10.1016/j.tins.2014.04.001, 24836381

[ref65] Ji J. L. Spronk M. Kulkarni K. Repovš G. Anticevic A. Cole M. W. (2019). Mapping the human brain’s cortical-subcortical functional network organization. NeuroImage 185, 35–57. doi: 10.1016/j.neuroimage.2018.10.006, 30291974 PMC6289683

[ref66] Kalafat M. Hugonot-Denier L. Poitrenaud J. (2003). Standardisation et étalonnage français du Mini Mental State (MMS) version GRÉCO. Rev. Neuropsychol. 13, 209–236.

[ref67] Kemper S. Herman R. E. Liu C.-J. (2004). Sentence production by young and older adults in controlled contexts. J. Gerontol. Ser. B Psychol. Sci. Soc. Sci. 59, P220–P224. doi: 10.1093/geronb/59.5.P220, 15358794

[ref68] Klimesch W. (2012). Alpha-band oscillations, attention, and controlled access to stored information. Trends Cogn. Sci. 16, 606–617. doi: 10.1016/j.tics.2012.10.007, 23141428 PMC3507158

[ref69] Kohl O. Woolrich M. Nobre A. C. Quinn A. (2023). Glasser52: A parcellation for MEG-Analysis [Jeu de données]. zenodo. doi: 10.5281/ZENODO.10401792

[ref70] Kosciessa J. Q. Mayr U. Lindenberger U. Garrett D. D. (2024). Broadscale dampening of uncertainty adjustment in the aging brain. Nat. Commun. 15:10717. doi: 10.1038/s41467-024-55416-2, 39715747 PMC11666723

[ref71] Krethlow G. Fargier R. Atanasova T. Ménétré E. Laganaro M. (2024). Asynchronous behavioral and neurophysiological changes in word production in the adult lifespan. Cereb. Cortex 34:bhae187. doi: 10.1093/cercor/bhae187, 38715409 PMC11077060

[ref72] Krishnan A. Williams L. J. McIntosh A. R. Abdi H. (2011). Partial least squares (PLS) methods for neuroimaging: a tutorial and review. NeuroImage 56, 455–475. doi: 10.1016/j.neuroimage.2010.07.034, 20656037

[ref73] Lee J. Man G. Keen A. Castro N. (2022). Priming sentence production in older adults: evidence for preserved implicit learning. Aphasiology 38, 1–21. doi: 10.1080/02687038.2022.2153326, 38425351 PMC10901520

[ref74] Lindenberger U. Baltes P. B. (1994). Sensory functioning and intelligence in old age: a strong connection. Psychol. Aging 9, 339–355. doi: 10.1037/0882-7974.9.3.339, 7999320

[ref75] Lindenberger U. Mayr U. (2014). Cognitive aging: is there a dark side to environmental support? Trends Cogn. Sci. 18, 7–15. doi: 10.1016/j.tics.2013.10.006, 24210962 PMC3969029

[ref76] Mantegna F. Poeppel D. Orpella J. (2026). Mu rhythm motor–auditory delay in imagined speech mirrors overt speech timing. Sci. Rep. 16:6528. doi: 10.1038/s41598-026-37421-1, 41606186 PMC12909796

[ref78] Mekkes N. J. Groot M. Hoekstra E. De Boer A. Dagkesamanskaia E. Bouwman S. . (2024). Identification of clinical disease trajectories in neurodegenerative disorders with natural language processing. Nat. Med. 30, 1143–1153. doi: 10.1038/s41591-024-02843-9, 38472295 PMC11031398

[ref79] Mihalik A. Chapman J. Adams R. A. Winter N. R. Ferreira F. S. Shawe-Taylor J. . (2022). Canonical correlation analysis and partial least squares for identifying brain–behavior associations: a tutorial and a comparative study. Biol. Psychiatry Cogn. Neurosci. Neuroimaging 7, 1055–1067. doi: 10.1016/j.bpsc.2022.07.012 35952973

[ref80] Mooraj Z. Salami A. Campbell K. L. Dahl M. J. Kosciessa J. Q. Nassar M. R. . (2025). Toward a functional future for the cognitive neuroscience of human aging. Neuron 113, 154–183. doi: 10.1016/j.neuron.2024.12.008, 39788085 PMC13032885

[ref81] Nigbur R. Ivanova G. Stürmer B. (2011). Theta power as a marker for cognitive interference. Clin. Neurophysiol. 122, 2185–2194. doi: 10.1016/j.clinph.2011.03.030, 21550845

[ref82] Oldfield R. C. (1971). The assessment and analysis of handedness: the Edinburgh inventory. Neuropsychologia 9, 97–113. doi: 10.1016/0028-3932(71)90067-4, 5146491

[ref83] Ozkalp-Poincloux B. Cassotti M. Salvia É. Doucet G. E. Camarda A. (2026). Older adults can outperform younger adults in creative problem solving. BMC Geriatr. 26. doi: 10.1186/s12877-025-06707-w, 41559572 PMC12903399

[ref84] Peelle J. E. (2019). “Language and aging,” in The Oxford Handbook of Neurolinguistics, ed. Peelle J. E. (Oxford: Oxford University Press), 294–316.

[ref85] Perrin F. Pernier J. Bertrand O. Echallier J. F. (1989). Spherical splines for scalp potential and current density mapping. Electroencephalogr. Clin. Neurophysiol. 72, 184–187. doi: 10.1016/0013-4694(89)90180-6, 2464490

[ref86] Pexman P. M. Diveica V. Binney R. J. (2023). Social semantics: the organization and grounding of abstract concepts. Philos. Trans. R. Soc. B 378:20210363. doi: 10.1098/rstb.2021.0363, 36571120 PMC9791475

[ref87] Piai V. Klaus J. Rossetto E. (2020). The lexical nature of alpha-beta oscillations in context-driven word production. J. Neurolinguistics 55:100905. doi: 10.1016/j.jneuroling.2020.100905

[ref88] Piai V. Roelofs A. Jensen O. Schoffelen J.-M. Bonnefond M. (2014). Distinct patterns of brain activity characterise lexical activation and competition in spoken word production. PLoS One 9:e88674. doi: 10.1371/journal.pone.0088674, 24558410 PMC3928283

[ref89] Piai V. Roelofs A. Rommers J. Maris E. (2015). Beta oscillations reflect memory and motor aspects of spoken word production. Hum. Brain Mapp. 36, 2767–2780. doi: 10.1002/hbm.22806, 25872756 PMC6869587

[ref90] Piai V. Zheng X. (2019). “Speaking waves: neuronal oscillations in language production,” in Psychology of Learning and Motivation, vol. 71. Ed. Federmeier K. D. (Elsevier), 265–302.

[ref91] Podvalny E. Noy N. Harel M. Bickel S. Chechik G. Schroeder C. E. . (2015). A unifying principle underlying the extracellular field potential spectral responses in the human cortex. J. Neurophysiol. 114, 505–519. doi: 10.1152/jn.00943.2014, 25855698 PMC4509389

[ref92] Price B. H. Gavornik J. P. (2022). Efficient temporal coding in the early visual system: existing evidence and future directions. Front. Comput. Neurosci. 16:929348. doi: 10.3389/fncom.2022.929348, 35874317 PMC9298461

[ref93] Pscherer C. Mückschel M. Beste C. (2026). Alpha band activity mediates age-related effects on three distinct aspects of working memory dynamics. Neurobiol. Aging 161, 1–13. doi: 10.1016/j.neurobiolaging.2026.01.001, 41529575

[ref94] Quinn A. J. Atkinson L. Z. Gohil C. Kohl O. Pitt J. Zich C. . (2024). The GLM-spectrum: a multilevel framework for spectrum analysis with covariate and confound modelling. Imaging Neurosci. 2, 1–26. doi: 10.1162/imag_a_00082, 40800496 PMC12224406

[ref96] Roger E. Banjac S. Thiebaut de Schotten M. Baciu M. (2022). Missing links: the functional unification of language and memory (L∪M). Neurosci. Biobehav. Rev. 133:104489. doi: 10.1016/j.neubiorev.2021.12.012, 34929226

[ref97] Roos N. M. Takashima A. Piai V. (2023). Functional neuroanatomy of lexical access in contextually and visually guided spoken word production. Cortex 159, 254–267. doi: 10.1016/j.cortex.2022.10.014, 36641964

[ref98] Rossi E. Diaz M. (2016). How aging and bilingualism influence language processing: theoretical and neural models. Linguist. Approach. Bilingualism 6, 9–42. doi: 10.1075/lab.14029.ros, 28919933 PMC5600288

[ref99] Smith S. M. (2002). Fast robust automated brain extraction. Hum. Brain Mapp. 17, 143–155. doi: 10.1002/hbm.10062, 12391568 PMC6871816

[ref100] Snaith R. P. Zigmond A. S. (1986). The hospital anxiety and depression scale. BMJ 292, 344.1–344.344. doi: 10.1136/bmj.292.6516.344, 3080166 PMC1339318

[ref101] Spitzer B. Haegens S. (2017). Beyond the status quo: a role for beta oscillations in endogenous content (re)activation. eNeuro 4:2017. doi: 10.1523/ENEURO.0170-17.2017, 28785729 PMC5539431

[ref102] Spreng R. N. Turner G. R. (2021). From exploration to exploitation: a shifting mental mode in late life development. Trends Cogn. Sci. 25, 1058–1071. doi: 10.1016/j.tics.2021.09.001, 34593321 PMC8844884

[ref103] Sung J. E. (2015). Age-related changes in sentence production abilities and their relation to working-memory capacity: evidence from a verb-final language. PLoS One 10:e0119424. doi: 10.1371/journal.pone.0119424, 25856161 PMC4391780

[ref104] Sung J. E. Jo E. Choi S. Lee J. (2024). Coordinating words and sentences: detecting age-related changes in language production. J. Speech Lang. Hear. Res. 67, 211–220. doi: 10.1044/2023_JSLHR-23-00222, 38099825 PMC11000805

[ref105] Tadel F. Baillet S. Mosher J. C. Pantazis D. Leahy R. M. (2011). Brainstorm: a user-friendly application for MEG/EEG analysis. Comput. Intell. Neurosci. 2011, 1–13. doi: 10.1155/2011/879716, 21584256 PMC3090754

[ref106] Taulu S. Simola J. (2006). Spatiotemporal signal space separation method for rejecting nearby interference in MEG measurements. Phys. Med. Biol. 51, 1759–1768. doi: 10.1088/0031-9155/51/7/008, 16552102

[ref107] Thiebaut de Schotten M. Forkel S. J. (2022). The emergent properties of the connected brain. Science 378, 505–510. doi: 10.1126/science.abq2591, 36378968

[ref108] Tombaugh T. (2004). Trail making test a and B: normative data stratified by age and education. Arch. Clin. Neuropsychol. 19, 203–214. doi: 10.1016/S0887-6177(03)00039-8, 15010086

[ref109] United Nations (2023). Leaving No One Behind in An Ageing World. United Nations: Department of Economic and Social Affairs at NYC.

[ref110] Van Es M. W. J. Higgins C. Gohil C. Quinn A. J. Vidaurre D. Woolrich M. W. (2025). Large-scale cortical functional networks are organized in structured cycles. Nat. Neurosci. 28, 2118–2128. doi: 10.1038/s41593-025-02052-8, 40866607 PMC12497652

[ref111] Van Veen B. D. Van Drongelen W. Yuchtman M. Suzuki A. (1997). Localization of brain electrical activity via linearly constrained minimum variance spatial filtering. IEEE Trans. Biomed. Eng. 44, 867–880. doi: 10.1109/10.623056, 9282479

[ref112] Vidaurre D. Hunt L. T. Quinn A. J. Hunt B. A. E. Brookes M. J. Nobre A. C. . (2018). Spontaneous cortical activity transiently organises into frequency specific phase-coupling networks. Nat. Commun. 9:2987. doi: 10.1038/s41467-018-05316-z, 30061566 PMC6065434

[ref113] Wei H. Power L. Singh K. D. Palaniyappan L. (2025). Beta oscillations as a mechanistic target for predictive processing deficits in psychosis. Biol. Life Sci. doi: 10.20944/preprints202511.2284.v1

[ref114] WHO (2025) Decade of healthy aging 2021-2030 Available online at: https://www.who.int/fr/initiatives/decade-of-healthy-ageing (Accessed June 22, 2026).

[ref115] Woolrich M. Hunt L. Groves A. Barnes G. (2011). MEG beamforming using Bayesian PCA for adaptive data covariance matrix regularization. NeuroImage 57, 1466–1479. doi: 10.1016/j.neuroimage.2011.04.041, 21620977 PMC4894461

[ref116] Yaghoubi M. Kumar M. G. Nieto-Posadas A. Mosser C.-A. Gisiger T. Wilson É. . (2026). Predictive coding of reward in the hippocampus. Nature 651, 414–420. doi: 10.1038/s41586-025-09958-0, 41535460

[ref117] Zheng X. Y. Piai V. (2025). Neural oscillations in the aging brain associated with interference control in word production. Neurobiol. Lang. 6:15. doi: 10.1162/nol.a.15, 41000093 PMC12459976

[ref118] Zioga I. Kenett Y. N. Giannopoulos A. Luft C. D. B. (2024). The role of alpha oscillations in free- and goal-directed semantic associations. Hum. Brain Mapp. 45:e26770. doi: 10.1002/hbm.26770, 38970217 PMC11226545

[ref119] Zioga I. Weissbart H. Lewis A. G. Haegens S. Martin A. E. (2023). Naturalistic spoken language comprehension is supported by alpha and beta oscillations. J. Neurosci. 43, 3718–3732. doi: 10.1523/JNEUROSCI.1500-22.2023, 37059462 PMC10198453

